# Early Relative eGFR Decline After SGLT2 Inhibitor Initiation in Heart Failure: Clinical Correlates and Pretreatment-Anchored 12-Month Trajectories

**DOI:** 10.3390/jcm15208158

**Published:** 2026-10-20

**Authors:** Yoriyasu Suzuki, Tomohiro Onishi, Yusuke Nakano, Masahiro Shimoda, Reiji Goto, Takahiko Suzuki, Tetsuya Amano

**Affiliations:** 1 Department of Cardiology, Aichi Medical University, Nagakute 480-1195, Aichi, Japan; oonishi.tomohiro.899@mail.aichi-med-u.ac.jp (T.O.); nakanoy@aichi-med-u.ac.jp (Y.N.);; 2 Department of Cardiology, Nagoya Heart Center, Nagoya 461-0045, Aichi, Japan; 3 Department of Cardiology, Toyohashi Heart Center, Toyohashi 441-8530, Aichi, Japan

**Keywords:** SGLT2 inhibitor, heart failure, estimated glomerular filtration rate, cardiorenal assessment, kidney function

## Abstract

**Background/Objectives:** Early changes in estimated glomerular filtration rate (eGFR) after sodium–glucose cotransporter 2 (SGLT2) inhibitor initiation in heart failure (HF) are difficult to interpret in routine practice. We assessed the clinical correlates of >10% early relative eGFR decline and the subsequent trajectories relative to the pretreatment eGFR. **Methods:** This retrospective single-center study included 295 patients with HF who remained on SGLT2 inhibitor treatment at their first eligible creatinine assessment on days 10–45 (median 25 days [IQR 21–30]; range 10–42). Multivariable regression, mixed-effects modeling, and within-patient paired analyses were used. **Results:** Among the 333 initiation episodes, 33 (9.9%) were excluded after early treatment discontinuation, and five repeat episodes were removed. Early decline >10% occurred in 110/295 patients (37.3%). Age ≥ 80 years (adjusted OR 2.14, 95% CI 1.21–3.81), female sex (2.51, 1.42–4.44), higher baseline eGFR per 10 mL/min/1.73 m^2^ (1.43, 1.21–1.69), and loop-diuretic use (1.82, 1.03–3.23) were associated with decline; the loop-diuretic association attenuated in an expanded model. Twelve-month eGFR was available for 195/295 patients (66.1%). Among the 71 with >10% early decline and paired 12-month data, the mean eGFR increased by 4.70 mL/min/1.73 m^2^ from the early assessment but remained 6.99 mL/min/1.73 m^2^ below the pretreatment (both *p* < 0.001). **Conclusions:** In this selected cohort, a mean increase after early decline did not indicate a return to the pretreatment mean. The interpretation of an isolated early eGFR value requires clinical context and subsequent measurements.

## 1. Introduction

Sodium–glucose cotransporter 2 (SGLT2) inhibitors are foundational therapies for heart failure (HF). Randomized trials have demonstrated fewer worsening-HF events across the left ventricular ejection fraction (LVEF) spectrum, while dedicated kidney trials have shown slower chronic kidney disease progression [1,2,3,4,5,6,7].

An initial fall in creatinine-derived estimated glomerular filtration rate (eGFR) is frequently observed after SGLT2 inhibitor initiation. In DAPA-HF, 38.2% of dapagliflozin-treated participants experienced a >10% relative reduction at day 14 [8]. A prespecified DELIVER analysis reported an early reduction >10% at 1 month in approximately 40% of dapagliflozin-treated patients, compared with 25% receiving a placebo [9]. EMPEROR and other studies have characterized similar changes in HF and related populations [10,11,12,13,14]. These observations support a possible hemodynamic component, but an individual creatinine-derived eGFR decline cannot be assumed to reflect an SGLT2-specific mechanism.

In HF, eGFR can also change with venous congestion, systemic perfusion, diuretic exposure, intercurrent illness, and adjustment of other HF therapies. Across previous studies, the timing and definition of the early assessment have differed: 14 days in DAPA-HF [8], 1 month in DELIVER [9], approximately 4 weeks in EMPEROR analyses [10,11], an early absolute decline in Ogata et al. [15], and up to 3 months in the broader cohort examined by Lee et al. [16]. The differences in the study populations and the ascertainment preclude a direct comparison of the reported frequencies or correlates.

The first creatinine measurement in routine care occurs at variable intervals and may reflect several concurrent clinical influences. A subsequent increase from an unusually low early measurement is also distinct from a restoration of the pretreatment value; selecting groups according to their baseline-to-early change introduces mathematical coupling and regression to the mean.

We therefore evaluated the frequency and clinical correlates of >10% early relative eGFR decline at the first eligible post-initiation assessment (days 10–45) in an older, real-world HF cohort. We also examined the observed eGFR through 12 months, combining adjusted longitudinal trajectories with paired comparisons anchored to each patient’s pretreatment value. This design was intended to characterize the observed measurements rather than establish a drug-specific renal mechanism or prognostic effect.

## 2. Materials and Methods

### 2.1. Study Design and Population

This retrospective, single-center observational study was performed at Nagoya Heart Center, Nagoya, Japan. Consecutive SGLT2 inhibitor initiation episodes in patients with HF were identified between 2020 and 2023; the latest initiation was on 31 October 2023. Initiation occurred during hospitalization in 150 patients (50.8%) and in the outpatient setting in 145 (49.2%). The degree of decompensation or stabilization at inpatient initiation was not consistently categorized. Study-center records were reviewed through 6 March 2025, one year after the last SGLT2 inhibitor prescription recorded at the center on 6 March 2024. Thus, all included patients had a calendar opportunity for at least 12 months of follow-up, irrespective of subsequent availability of laboratory results.

**Figure 1:**
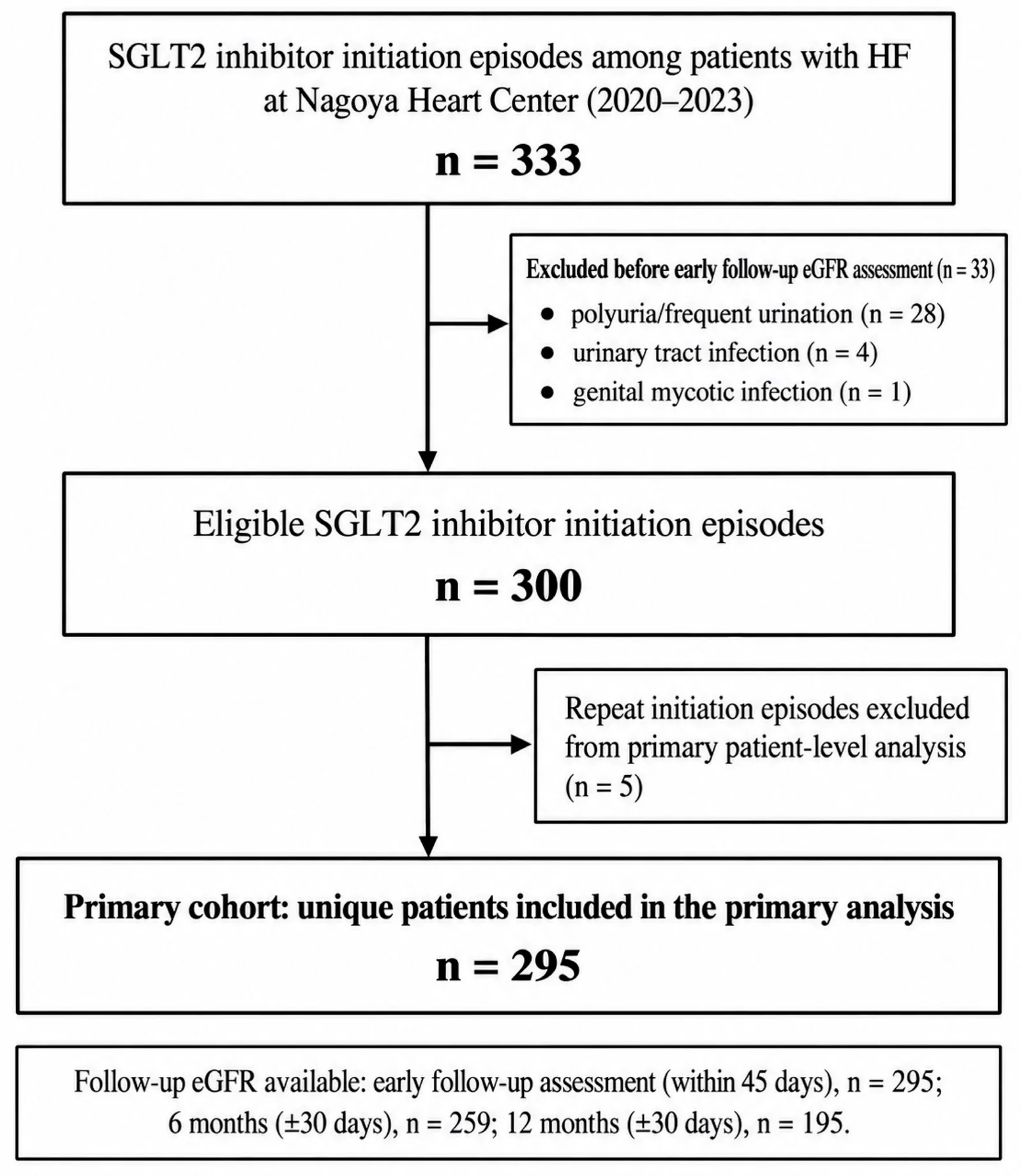
Study flowchart. Among 333 SGLT2 inhibitor initiation episodes, 33 were excluded following discontinuation before early reassessment and five additional repeat episodes were excluded to provide one observation per patient. The final analytic cohort comprised 295 unique patients. The selected early assessment occurred 10–42 days after initiation (median 25 days [IQR 21–30]); a total of 259 and 195 patients contributed eGFR measurements at approximately 6 and 12 months, respectively. Counts refer to initiation episodes until the final patient-level cohort is defined.

Of 333 identified initiation episodes, 33 (9.9%), corresponding to 33 distinct patients without overlap, were excluded because SGLT2 inhibitor therapy had been discontinued before early reassessment, owing to polyuria or frequent urination (n = 28), urinary tract infection (n = 4), or genital mycotic infection (n = 1). The remaining 300 qualifying initiation episodes had pretreatment and early eGFR measurements. Five were repeat initiation episodes in patients already represented; retaining the earliest qualifying episode yielded 295 unique patients (Figure 1). Accordingly, this selected cohort represents patients with documented treatment continuation and early laboratory reassessment rather than all patients who initiated an SGLT2 inhibitor.

At observed eGFR assessments included in the longitudinal dataset, SGLT2 inhibitor treatment was documented as ongoing; measurements after documented discontinuation were not included. For patients referred back to their primary care physicians, treatment continuation after referral was confirmed by telephone follow-up. Such confirmation did not, however, provide creatinine measurements from outside the study center or establish uninterrupted exposure at every intervening time point. HF was diagnosed by treating physicians according to the Universal Definition and Classification of Heart Failure [17], based on a compatible clinical syndrome with objective evidence of cardiac structural or functional abnormality and elevated natriuretic peptides and/or congestion. Patients were classified as HFrEF (LVEF ≤ 40%), HFmrEF (41–49%), or HFpEF (≥50%); HFpEF was not diagnosed from LVEF alone. The echocardiography-to-initiation interval was not standardized. Choice of SGLT2 inhibitor and background HF therapy followed routine care.

This study was approved by the Institutional Review Board of Nagoya Heart Center (Approval No. 2023-015) and conducted in accordance with the Declaration of Helsinki. Written informed consent was waived because of the retrospective design, and an opt-out approach was used where applicable.

### 2.2. Data Collection and Definitions

Demographic, clinical, laboratory, echocardiographic, and medication data were obtained from medical records. Baseline variables included age, sex, body mass index (BMI), hypertension, diabetes mellitus, New York Heart Association (NYHA) functional class, HF phenotype/LVEF, N-terminal pro-B-type natriuretic peptide (NT-proBNP), and concomitant treatment. Baseline medication exposure was defined at SGLT2 inhibitor initiation. Renin–angiotensin system (RAS) inhibitor use included angiotensin-converting enzyme inhibitors and angiotensin receptor blockers but excluded angiotensin receptor–neprilysin inhibitors (ARNIs), which were classified separately. ARNI exposure was categorized as no recorded use, use already established at SGLT2 inhibitor initiation, or initiation after SGLT2 inhibitor treatment (Table S7). Accordingly, the absence of ARNI did not imply absence of other RAS inhibition. Subsequent medication titration and switching were not comprehensively characterized.

Creatinine-derived eGFR was calculated using the Japanese equation [18]: eGFR (mL/min/1.73 m^2^) = 194 × serum creatinine (mg/dL)^−1.094 × age (years)^−0.287 × 0.739 (if female). The same serum creatinine assay method was used throughout the study period. Pretreatment creatinine was selected from samples obtained within 10 days before the first SGLT2 inhibitor dose; if multiple results were available, the sample closest to initiation was used, with same-day sampling performed before dosing. Early eGFR was the first available post-initiation measurement from day 10 through day 45 (inclusive); measurements obtained on days 1–9 were not eligible for the early assessment (observed range, 10–42 days; median 25 [IQR 21–30] days). For assessments at approximately 6 and 12 months (±30 days), the sample closest to the target date was selected if multiple results were available. These were the original data-selection rules, applied identically to both early-decline groups.

Early relative eGFR change (%) was defined as 100 × (early eGFR − pretreatment eGFR)/pretreatment eGFR; negative values indicate decline. The primary binary outcome was a >10% relative reduction (change <−10%), an operational threshold facilitating comparison with prior HF studies rather than a definition of hemodynamic dip, acute kidney injury (AKI), or structural renal injury. Absolute decline (pretreatment minus early eGFR; mL/min/1.73 m^2^) was analyzed as a continuous outcome. Exploratory thresholds of >15% and >20%, descriptive frequency of >30% decline, and pretreatment eGFR strata of <45, 45–<60, and ≥60 mL/min/1.73 m^2^ were also examined.

### 2.3. Study Outcomes

The primary objective was to identify baseline clinical correlates of >10% early relative eGFR decline. Secondary analyses characterized observed eGFR through 12 months by early-decline category. In patients with the corresponding measurements, paired analyses quantified both early-to-follow-up and pretreatment-to-follow-up changes within individuals. The 6- and 12-month paired analyses were based on separately available subsets and were not interpreted as a single complete longitudinal panel.

### 2.4. Statistical Analysis

Continuous variables are reported as mean ± standard deviation or median [interquartile range], and categorical variables as n (%). Between-group comparisons used Welch’s *t* test, the Mann–Whitney U test for NT-proBNP, and chi-square or Fisher’s exact tests as appropriate. These comparisons were descriptive and were not used to select multivariable predictors.

A multivariable logistic regression model assessed >10% early decline using nine clinically selected baseline covariates: age ≥ 80 years, female sex, pretreatment eGFR (per 10 mL/min/1.73 m^2^), ARNI in use at SGLT2 inhibitor initiation, loop-diuretic use, diabetes mellitus, HFpEF, tolvaptan use, and BMI (per 5 kg/m^2^). The primary model comprised 295 complete observations, with no covariate imputation. Adjusted odds ratios (ORs), Wald 95% confidence intervals (CIs), and two-sided *p*-values are presented. The model estimated associations rather than causal drug effects or individual risk predictions; no formal sample-size calculation was performed.

Repeated eGFR measurements at the early, 6-month, and 12-month assessments were analyzed using a linear mixed-effects model with categorical time, early-decline group, their interaction, baseline eGFR, age ≥ 80 years, female sex, baseline ARNI use, and loop-diuretic use as fixed effects and a patient-specific random intercept. The model was fitted by restricted maximum likelihood (REML); the early assessment was the reference time, and pretreatment eGFR was an adjustment covariate rather than a repeated outcome. Adjusted marginal means were estimated using the emmeans package, standardized to the corresponding covariate means of the 295 unique patients. Model-based 95% CIs and two-sided *p*-values for prespecified within- and between-group contrasts were obtained from the fitted model using emmeans, without multiplicity adjustment. The overall group-by-time interaction was assessed by a likelihood-ratio test comparing nested models refitted by maximum likelihood (ML; 2 degrees of freedom). Since early-decline status was defined relative to pretreatment eGFR, the trajectories were interpreted in light of mathematical coupling and regression to the mean.

All 295 patients contributed an early eGFR measurement, 259 contributed a 6-month value, and 195 contributed a 12-month value (749 observations). Study-center records were available through 6 March 2025, providing every included patient the calendar opportunity for 12-month assessment. After referral back to primary care, continued SGLT2 inhibitor treatment was confirmed by telephone; nevertheless, some patients did not return for creatinine testing at the study center, and outside laboratory results were unavailable for the analysis. The numbers attributable specifically to referral and other reasons for absent measurements were not systematically recorded. Missing eGFR values were not imputed. Mixed-effects inference assumes missingness at random conditional on observed information, which cannot be verified. Characteristics by 12-month measurement availability were compared (Table S1), and the mixed-effects analysis was repeated in patients with 12-month data (Table S4B). Within-patient contrasts used two-sided paired *t* tests.

An expanded logistic model additionally included NYHA class III/IV, mineralocorticoid receptor antagonist (MRA) use, and SGLT2 inhibitor type to examine sensitivity to indicators of HF severity and treatment intensity (Table S5). Baseline ARNI exposure referred only to use at SGLT2 inhibitor initiation; later ARNI initiation was described separately (Table S7). In the absence of randomized treatment assignment or complete drug-titration histories, medication coefficients were considered exploratory associations rather than comparative effects.

Sensitivity analyses comprised multivariable linear regression of continuous absolute early eGFR decline with HC3 heteroskedasticity-robust standard errors; alternative relative-decline thresholds (>15% and >20%); baseline eGFR strata; exclusion of six extreme early changes; generalized estimating equations (GEEs) with an exchangeable working correlation structure and sandwich-robust standard errors; a mixed-effects analysis restricted to patients with 12-month data; age entered continuously and a four-knot restricted cubic spline for baseline eGFR; and models incorporating the time to early creatinine measurement (Tables S2–S8). GEE-adjusted marginal means were standardized to the covariate means of the 295-patient cohort; the restricted mixed-effects analysis was standardized to the corresponding means of its 195-patient subset. Spline nonlinearity was evaluated by a likelihood-ratio test comparing spline and linear models. Exploratory comparisons were not adjusted for multiplicity. Analyses were conducted in R (version 4.6.1; Foundation for Statistical Computing, Vienna, Austria) using the lme4, emmeans, geepack, and sandwich packages, with EZR version 1.70 used as an R-based statistical interface [19].

ChatGPT (OpenAI, GPT-6) was used to assist with English-language drafting and editing, manuscript organization, and the preparation of responses to reviewers. The tool was not used for patient-level data processing or statistical calculations. All statistical analyses were independently performed and verified by the authors using R, and all AI-assisted content, references, and scientific interpretations were critically reviewed by the authors.

## 3. Results

### 3.1. Study Population and Baseline Characteristics

Of the 333 SGLT2 inhibitor initiation episodes, 33 (9.9%), representing 33 distinct patients, were excluded after discontinuation before early reassessment; five repeat episodes were subsequently removed, yielding 295 unique patients (Figure 1). Early creatinine sampling occurred 10–42 days after initiation (median 25 [IQR 21–30] days): five patients were assessed at 10–13 days, 61 at 14–20 days, 134 at 21–29 days, and 95 at 30–45 days (Table S6). Eligible eGFR measurements were available for 259 patients (87.8%) at 6 months and 195 (66.1%) at 12 months; 100 (33.9%) lacked a 12-month measurement despite sufficient calendar time for a follow-up. Referral back to primary care without subsequent study-center testing contributed to missing laboratory data, although the individual reasons could not be assigned reliably to all of the patients.

The mean age was 74.6 ± 11.3 years, 89 patients (30.2%) were female, and the mean pretreatment eGFR was 54.8 ± 17.4 mL/min/1.73 m^2^. HFrEF, HFmrEF, and HFpEF were present in 91 (30.8%), 54 (18.3%), and 150 (50.8%) patients, respectively; 150 (50.8%) initiated SGLT2 inhibitor treatment as inpatients. Compared with the ≤10% early-decline group, those with >10% decline had a higher proportion of women (40.9% vs. 23.8%), a higher pretreatment eGFR (58.1 ± 17.4 vs. 52.8 ± 17.1 mL/min/1.73 m^2^), and more NYHA III/IV symptoms, MRA use, loop-diuretic use, and inpatient initiation (Table 1). The baseline ARNI use was similar (18.2% vs. 16.2%; *p* = 0.784). At initiation, 77 patients used an ACE inhibitor or an ARB as a separate RAS-inhibitor category; among the 169 with no ARNI recorded, 76 were receiving an ACE inhibitor or an ARB.

Among the 195 patients with an available 12-month eGFR, 71 (36.4%) had experienced >10% early decline, compared with 39/100 (39.0%) without a 12-month value (*p* = 0.703). Loop-diuretic use was more frequent in the patients with a 12-month measurement (63.1% vs. 44.0%; *p* = 0.002; Table S1). Thus, although absent measurements were not due to insufficient calendar follow-up and medication continuation was checked after referral, the observed longitudinal cohort could still be selected on clinical or treatment characteristics. These comparisons and complete-case sensitivity analyses cannot rule out informative missingness.

### 3.2. Frequency and Clinical Correlates of Early eGFR Decline

**Figure 2:**
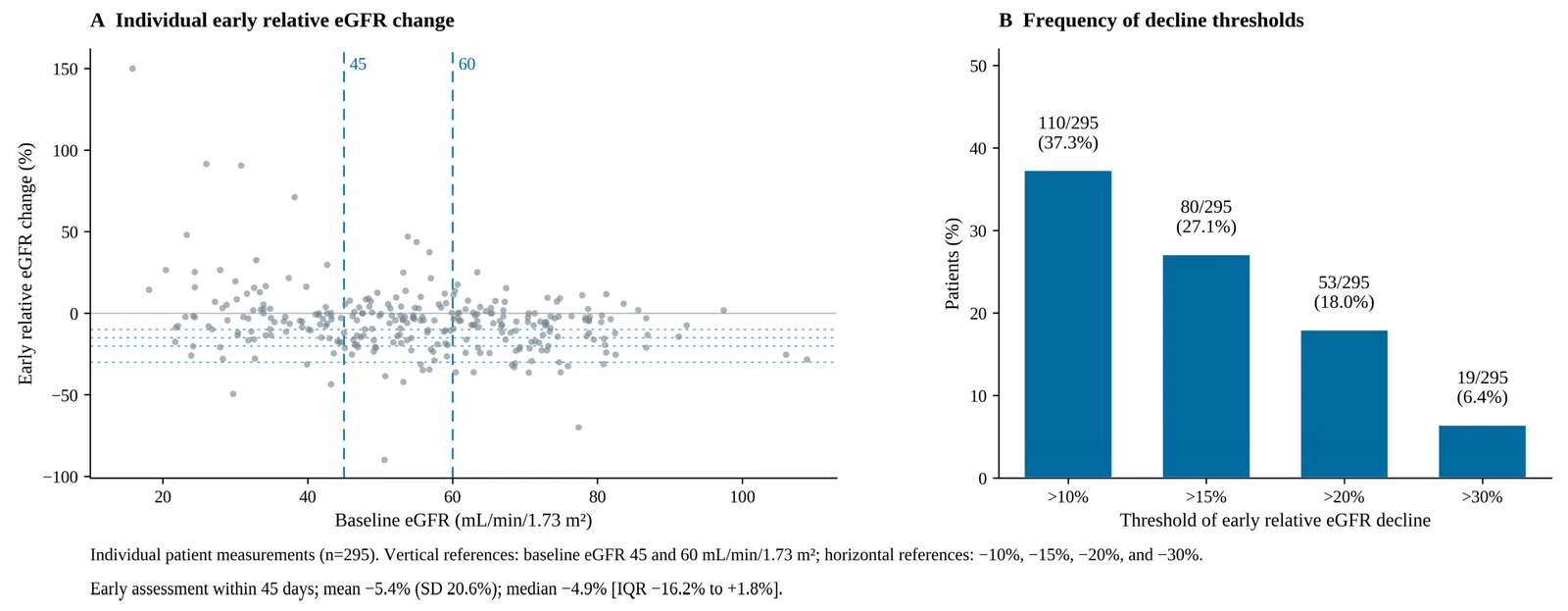
Early relative eGFR change at the first eligible post-initiation assessment (days 10–45). (**A**) Individual relative changes plotted against pretreatment eGFR; reference lines indicate baseline eGFR values of 45 and 60 mL/min/1.73 m^2^ and decline thresholds of 10%, 15%, 20%, and 30%. (**B**) Frequencies of patients exceeding each decline threshold (n = 295). Measurements were obtained 10–42 days after initiation (median 25 [IQR 21–30] days). Early relative change was mean −5.4% (SD 20.6%) and median −4.9% [IQR −16.2% to +1.8%]. Baseline eGFR occurs in the denominator of relative change; the association depicted in Panel A is therefore descriptive and susceptible to mathematical coupling.

At the first eligible post-initiation assessment, 110/295 patients (37.3%) had >10% relative eGFR decline. The mean relative change was −5.4% (SD 20.6%), and the median change was −4.9% [IQR −16.2% to +1.8%]. Declines of >15%, >20%, and >30% occurred in 80 (27.1%), 53 (18.0%), and 19 (6.4%) patients, respectively (Figure 2). As the sampling occurred over 10–42 days, the observed 37.3% is not the incidence of decline at a common fixed post-initiation time.

**Figure 3:**
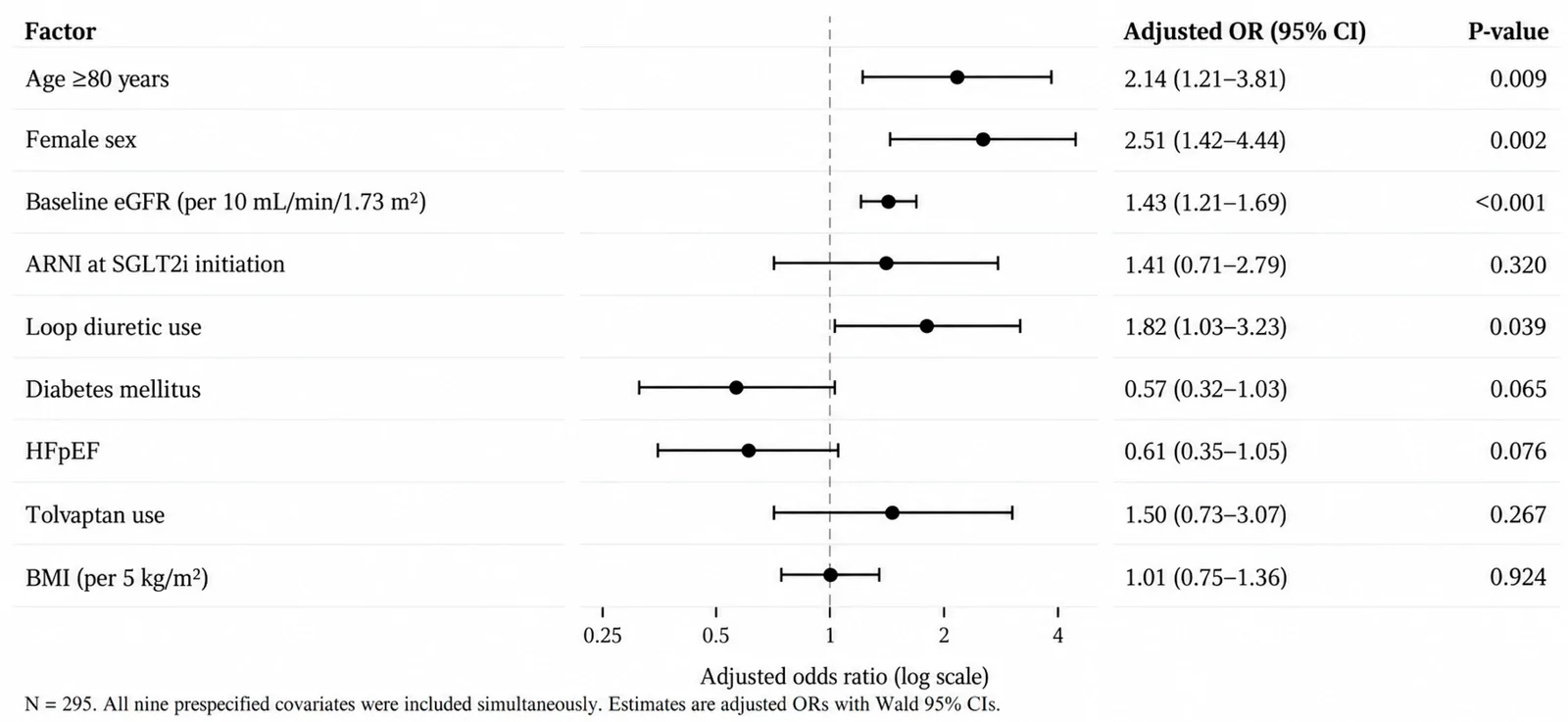
Multivariable clinical correlates of >10% early relative eGFR decline. Forest plot of adjusted ORs and 95% CIs from the primary logistic model in 295 unique patients. The nine concurrently modeled covariates were age ≥ 80 years, female sex, baseline eGFR (per 10 mL/min/1.73 m^2^), ARNI already in use at SGLT2 inhibitor initiation, loop-diuretic use, diabetes mellitus, HFpEF, tolvaptan, and BMI (per 5 kg/m^2^). These associations are observational and should not be interpreted as evidence of a causal drug-specific eGFR response.

In the nine-covariate logistic model (Figure 3), age ≥ 80 years (adjusted OR 2.14, 95% CI 1.21–3.81; *p* = 0.009), female sex (2.51, 1.42–4.44; *p* = 0.002), higher pretreatment eGFR per 10 mL/min/1.73 m^2^ (1.43, 1.21–1.69; *p* < 0.001), and loop-diuretic use (1.82, 1.03–3.23; *p* = 0.039) were associated with >10% early decline. Baseline ARNI exposure showed no clear association (1.41, 0.71–2.79; *p* = 0.320). In the complementary continuous model, an absolute early decline was greater with age ≥ 80 years (adjusted β +3.65 mL/min/1.73 m^2^, 95% CI +1.46 to +5.84), female sex (+4.94, +2.25 to +7.63), and a higher pretreatment eGFR per 10 mL/min/1.73 m^2^ (+2.61, +1.92 to +3.31; all *p* ≤ 0.001); the ARNI estimate was imprecise (+1.35, −1.46 to +4.16; *p* = 0.346; Table S2A). The continuous approach avoids a single dichotomous cutoff but does not eliminate confounding or baseline-related mathematical dependence.

### 3.3. Subsequent eGFR Trajectories and Paired Analyses

**Figure 4:**
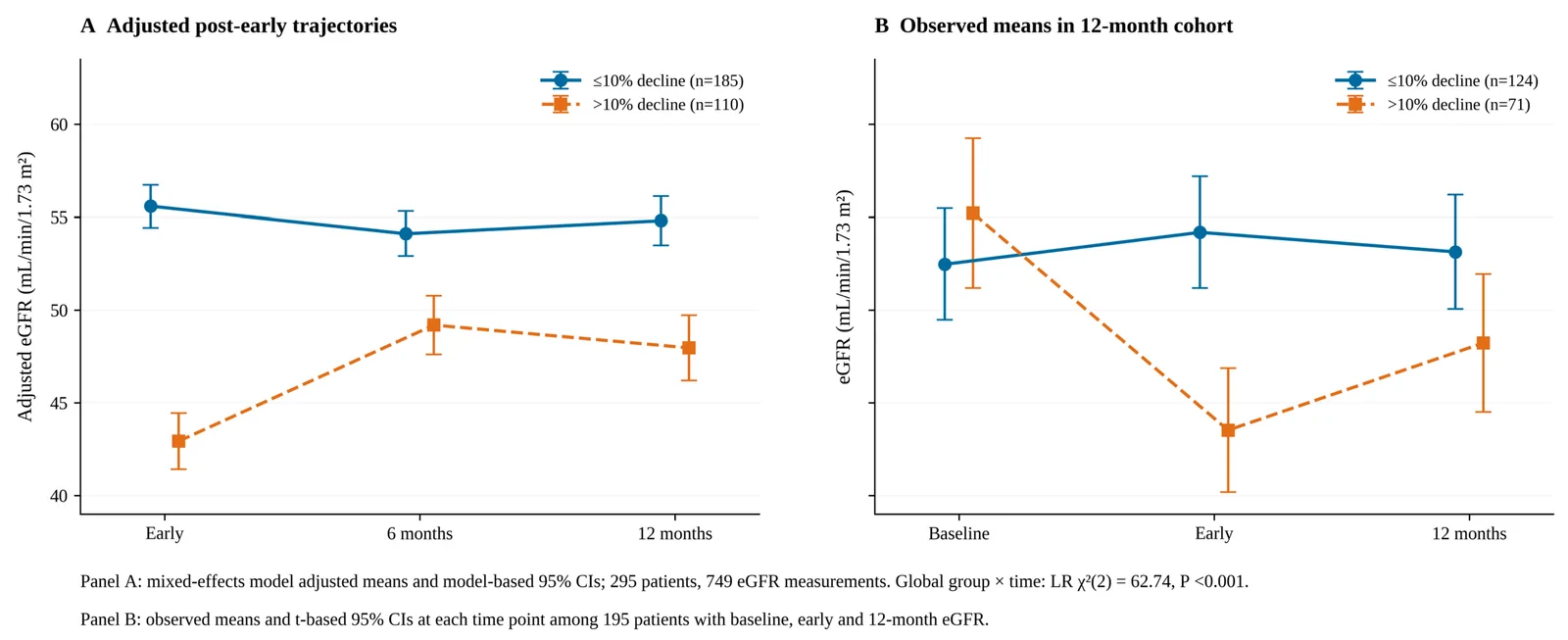
eGFR trajectories by early-decline classification. (**A**) Adjusted marginal mean eGFR and model-based 95% CIs after the first post-initiation assessment at 6 and 12 months, from the mixed-effects model adjusted for baseline eGFR, age ≥ 80, female sex, ARNI use at initiation, and loop-diuretic use. Early group n = 185/110 (≤10%/>10%), 6-month n = 163/96, 12-month n = 124/71. (**B**) Observed means and 95% CIs at pretreatment baseline, early assessment, and 12 months among 195 patients with paired values (n = 124/71). Since early groups were defined by a baseline-to-early change, the observed subsequent increase is intrinsically vulnerable to regression to the mean and does not demonstrate biological reversal of kidney injury or that each patient recovered to baseline.

The mixed-effects analysis included 749 eGFR observations from 295 patients (Figure 4A). A group-by-time interaction was observed (*p* < 0.001), although its magnitude is intrinsically influenced by defining groups according to the preceding baseline-to-early change. In the patients with >10% early decline, the adjusted mean eGFR increased from the early assessment by 6.26 mL/min/1.73 m^2^ at 6 months (95% CI 4.72–7.80) and 5.01 at 12 months (3.30–6.73; both *p* < 0.001). The corresponding changes in the ≤10% group were −1.46 (−2.64 to −0.28; *p* = 0.015) and −0.77 (−2.07 to +0.54; *p* = 0.250). The between-group differences in change were +7.72 at 6 months and +5.78 mL/min/1.73 m^2^ at 12 months (both *p* < 0.001). These estimates describe the observed post-early trajectories and must not be interpreted as evidence of renal tissue recovery.

At 6 months, paired data were available for 259 patients (≤10% decline, n = 163; >10% decline, n = 96); at 12 months, a different paired subset of 195 patients was available (n = 124 and n = 71; Table 2). In the >10% group with 6-month data, the mean eGFR increased by 5.94 mL/min/1.73 m^2^ from the early value (95% CI +4.38 to +7.51; *p* < 0.001) but remained 5.84 below the pretreatment (−7.55 to −4.12; *p* < 0.001). Among the 71 patients with paired 12-month data, the mean eGFR increased by 4.70 (+2.95 to +6.45; *p* < 0.001) from the early assessment but remained 6.99 below the pretreatment (−9.20 to −4.78; *p* < 0.001; Figure 4B). In the ≤10% group, the mean pretreatment-to-follow-up differences were +0.28 at 6 months (*p* = 0.698) and +0.67 at 12 months (*p* = 0.457). Since the 6- and 12-month analyses use different available-case subsets, they do not provide a direct paired comparison between those two follow-up times. Group means do not establish each patient’s recovery status.

### 3.4. Sensitivity Analyses

The sensitivity analyses broadly supported the descriptive findings while demonstrating a dependence on the model definition (Tables S2–S8). Older age, female sex, and higher pretreatment eGFR were associated with larger continuous absolute declines (Table S2A); female sex and pretreatment eGFR remained associated across the >15% and >20% thresholds (Table S2B). Nineteen patients exceeded the >30% threshold, too few for a stable nine-covariate model. The frequencies of >10% decline were 24/86 (27.9%), 39/92 (42.4%), and 47/117 (40.2%) in the pretreatment eGFR strata <45, 45–<60, and ≥60 mL/min/1.73 m^2^ (Table S2C). The exclusion of six extreme changes did not materially alter the main associations (Table S3).

The GEE showed a similar post-early trajectory (group-by-time Wald χ^2^ = 58.14, df = 2; *p* < 0.001; Table S4A), and the mixed-effects analysis restricted to the 195 patients with 12-month data was directionally consistent (Table S4B); neither excludes bias from missing outcomes. In the expanded logistic model, the loop-diuretic OR decreased from 1.82 to 1.18 (95% CI 0.62–2.24; *p* = 0.621), while MRA use was associated with a decline (OR 1.87, 1.02–3.43; *p* = 0.044); the baseline ARNI remained without a clear association (OR 1.79, 0.81–3.95; *p* = 0.147; Table S5). A longer time to the first creatinine test was associated with a decline (OR 1.48 per 7 days, 1.10–1.99; *p* = 0.009). After the inclusion of the sampling interval, the estimated OR for the baseline ARNI was 1.00 (0.48–2.07), compared with 1.41 in the primary model (Table S6), emphasizing sensitivity to the assessment timing rather than a causal effect. When age was modeled continuously, the OR per decade was 1.45 (1.11–1.90; *p* = 0.007), and the spline comparison did not detect nonlinearity in the baseline eGFR (likelihood-ratio χ^2^ = 1.39, df = 2; *p* = 0.499; Table S8).

## 4. Discussion

In this selected, single-center HF cohort, 37.3% of the patients receiving an SGLT2 inhibitor at the eligible 10–45-day reassessment had a >10% relative eGFR decline. Older age, female sex, and a higher pretreatment eGFR were consistently associated with the early measurement change; the associations with concomitant HF therapy depended on the model specification. The principal descriptive finding is that, among the patients with paired follow-up measurements, the mean eGFR rose from the early assessment but remained below the observed pretreatment mean. Neither the magnitude of the initial change nor a subsequent movement establishes its pharmacologic mechanism or clinical prognosis.

Early eGFR decline after SGLT2 inhibitor treatment has been examined in DAPA-HF, DELIVER, and EMPEROR [8,9,10,11]. The proportions reported in DAPA-HF and DELIVER were numerically close to that in our cohort, but differences in time points, clinical setting, selection after treatment initiation, and randomized comparison groups limit direct interpretation. DELIVER found no evidence that an early decline with dapagliflozin conferred a higher subsequent cardiovascular or renal risk [9]; our study did not measure those outcomes. Its contribution is therefore additional observational evidence from an older HF population spanning the LVEF spectrum, with mixed inpatient/outpatient initiation and paired comparisons anchored to the measured pretreatment eGFR—not identification of a new drug-specific mechanism or a clinical treatment effect.

Older age, female sex, and a higher pretreatment eGFR were associated with an early decline, but these observations require a cautious interpretation. The Japanese creatinine-based eGFR equation contains age and sex terms; in a within-person relative eGFR ratio, the fixed sex multiplier cancels, as does the age factor when the same age is used for measurements obtained a few weeks apart. Their observed associations are therefore not explained solely by the inclusion of those terms in the equation. Nonetheless, variation in creatinine generation, underlying clinical characteristics, and unmeasured confounding can affect the estimates. In contrast, absolute eGFR changes retain the equation’s scaling factors. The pretreatment eGFR is also part of the relative-change outcome and is vulnerable to mathematical coupling and regression to the mean. The different direction reported by Lee et al. [16] may reflect differences in populations, assessment intervals, and endpoint definitions; the present data cannot distinguish among these possibilities.

The interpretation of drug-related correlates is especially sensitive to the exposure window. At SGLT2 inhibitor initiation, 77 patients were receiving an ACE inhibitor or ARB therapy, which was analyzed separately from an ARNI. Of the 169 patients with no ARNI recorded, 76 were receiving an ACE inhibitor or an ARB; thus, the no-ARNI category does not represent an untreated RAS-system comparator. Fifty patients had an ARNI already recorded at SGLT2 inhibitor initiation, whereas 76 started an ARNI later (median 4 days [IQR 3–5] afterward; Table S7). Among the latter, 67/76 (88.2%) had initiated SGLT2 inhibition during hospitalization, compared with 1/50 (2.0%) among the baseline ARNI users. These imbalances, contemporaneous HF treatment changes, and incomplete switching/titration histories constrain the inference about ARNI-specific effects. A baseline ARNI use was not associated with early decline in the primary model, and its OR changed from 1.41 to 1.00 when early sampling time was considered (Table S6). Similarly, the loop-diuretic association attenuated after an additional adjustment for NYHA class, MRA, and SGLT2 inhibitor type (Table S5). These associations should not be interpreted as combined drug-induced renal effects.

The pretreatment-anchored paired comparisons provide the clearest longitudinal clinical description. The patients were categorized by a large baseline-to-early decline, so some subsequent increase is expected through regression to the mean, even in the absence of biological improvement. In the 71 patients with >10% early decline and 12-month paired data, the mean eGFR was 4.70 mL/min/1.73 m^2^ above the early assessment but 6.99 below the pretreatment mean. This mean deficit does not imply that every individual failed to return to baseline, nor does a single pretreatment measurement establish an untreated counterfactual. A decline arising from hemodynamic changes cannot be distinguished from AKI or other causes using the available creatinine data alone.

The analysis of absolute eGFR decline as a continuous outcome reduced the dependence on the >10% threshold but did not remove the baseline-related mathematical dependence. Similarly, the agreement between the mixed-effects and the GEE results supports a consistent descriptive pattern among the observed measurements, not a correction of attrition bias. The evidence from a meta-analysis of chronic SGLT2 inhibitor use and contrast-associated AKI in a different setting [20] illustrates why creatinine changes and structural renal injury should not be equated; it cannot identify the mechanism of an individual decline in this cohort.

In practice, an isolated early eGFR result after SGLT2 inhibitor initiation should be considered alongside congestion and volume status, blood pressure, intercurrent illness, concurrent HF medication changes, and subsequent renal measurements. Neither incomplete restoration of the pretreatment mean nor an early >10% decline in this observational dataset provides a basis to stop SGLT2 inhibitor therapy, define a surveillance interval, or estimate the cardiovascular or kidney prognosis. Treatment decisions should be informed by the broader randomized evidence and the individual clinical context [8,9].

Several limitations merit emphasis. First, 33 of the 333 initiation episodes (9.9%), corresponding to distinct patients, were excluded after early treatment discontinuation; the observed 37.3% frequency therefore pertains to the patients still receiving treatment who had an eligible early test, not all initiators. Second, the early assessment timing varied from 10 to 42 days, and longer intervals were associated with a higher probability of meeting the relative-decline threshold (Table S6). The variable sampling schedules and the concurrent HF treatment changes preclude a mechanistic attribution, even though creatinine testing used the same assay method throughout. Third, all the included patients had at least 12 months of potential follow-up through 6 March 2025, but 100/295 (33.9%) lacked an eligible 12-month creatinine measurement at the study center. After referral to primary care, SGLT2 inhibitor continuation was checked by telephone; outside laboratory results were nevertheless unavailable, and the individual reasons for missing measurements were not systematically coded. Those with 12-month data were more likely to use loop diuretics (63.1% vs. 44.0%), so informative missingness remains possible. Neither the mixed-effects modeling nor the sensitivity analyses eliminate this uncertainty. Fourth, the absence of an untreated comparator, a nonrandomized concomitant therapy, and incomplete serial data on hemodynamics, congestion, albuminuria, drug titration, and adjudicated AKI preclude assigning any observed change to SGLT2 inhibition alone. Fifth, creatinine-derived eGFR is affected by non-GFR determinants, and categorization using a pretreatment-referenced change entails mathematical coupling and regression to the mean. Finally, cardiovascular events, HF hospitalizations, death, and kidney outcomes were not assessed; no prognostic or treatment-decision claims can be made.

## 5. Conclusions

Among the selected patients with HF assessed during ongoing SGLT2 inhibitor treatment, 37.3% experienced a >10% relative eGFR decline at their first early measurement. In the subgroup with paired 12-month data, the mean eGFR increased from the early assessment but remained below the observed pretreatment mean. These findings are descriptive and do not establish an SGLT2-specific mechanism, uniform individual recovery, or prognostic significance. Early eGFR changes should be interpreted with clinical status, concomitant treatment, and subsequent measurements.

## Figures and Tables

**Table 1:** Baseline characteristics of the primary cohort.

Variable	Overall (N = 295)	≤10% Decline (n = 185)	>10% Decline (n = 110)	*p*-Value
Age, years	74.6 ± 11.3	73.6 ± 11.3	76.2 ± 11.3	0.05
Age ≥ 80 years	108 (36.6%)	60 (32.4%)	48 (43.6%)	0.071
Female sex	89 (30.2%)	44 (23.8%)	45 (40.9%)	0.003
BMI, kg/m^2^	24.0 ± 4.3	24.0 ± 3.9	23.9 ± 4.8	0.754
Baseline eGFR, mL/min/1.73 m^2^	54.8 ± 17.4	52.8 ± 17.1	58.1 ± 17.4	0.012
LVEF, %	48.2 ± 16.3	48.8 ± 16.1	47.4 ± 16.5	0.472
HFrEF	91 (30.8%)	55 (29.7%)	36 (32.7%)	0.632
HFmrEF	54 (18.3%)	32 (17.3%)	22 (20.0%)	
HFpEF	150 (50.8%)	98 (53.0%)	52 (47.3%)	
NYHA class III/IV	142 (48.1%)	78 (42.2%)	64 (58.2%)	0.011
NT-proBNP, pg/mL	1399.0 [834.5–2876.5]	1223.0 [727.0–2518.0]	1628.5 [942.0–3187.8]	0.097
Hypertension	157 (53.2%)	97 (52.4%)	60 (54.5%)	0.817
Diabetes mellitus	83 (28.1%)	59 (31.9%)	24 (21.8%)	0.084
RAS inhibitor use (excluding ARNI)	77 (26.1%)	54 (29.2%)	23 (20.9%)	0.153
ARNI at SGLT2i initiation	50 (16.9%)	30 (16.2%)	20 (18.2%)	0.784
Beta-blocker use	249 (84.4%)	154 (83.2%)	95 (86.4%)	0.583
MRA use	171 (58.0%)	93 (50.3%)	78 (70.9%)	<0.001
Loop-diuretic use	167 (56.6%)	95 (51.4%)	72 (65.5%)	0.025
Tolvaptan use	54 (18.3%)	32 (17.3%)	22 (20.0%)	0.671
Dapagliflozin	171 (58.0%)	97 (52.4%)	74 (67.3%)	0.018
Empagliflozin	124 (42.0%)	88 (47.6%)	36 (32.7%)	
Inpatient SGLT2i initiation	150 (50.8%)	83 (44.9%)	67 (60.9%)	0.011

Data are presented as mean ± SD, median [IQR], or n (%). *p*-values were calculated using Welch’s *t* test for continuous variables, the Mann–Whitney U test for NT-proBNP, and Yates-corrected chi-square tests for binary variables, with Fisher’s exact test used when expected cell counts were <5. The *p*-value for HF phenotype represents the overall comparison of HFrEF, HFmrEF, and HFpEF using Pearson’s chi-square test. Early eGFR decline >10% was defined relative to baseline eGFR at the first eligible assessment on days 10–45 of SGLT2 inhibitor initiation. ARNI exposure was defined as use at SGLT2 inhibitor initiation.

**Table 2:** Paired changes in eGFR at 6 and 12 months.

Follow-Up	Early Decline	n	Baseline eGFR	Early eGFR	Follow-Up eGFR	Early → Follow-Up Δ (95% CI)	*p*-Value	Baseline → Follow-Up Δ (95% CI)	*p*-Value
6 months	≤10% decline	163	52.16	53.98	52.44	−1.54 (−2.81 to −0.27)	0.018	+0.28 (−1.15 to +1.71)	0.698
6 months	>10% decline	96	57.02	45.24	51.19	+5.94 (+4.38 to +7.51)	<0.001	−5.84 (−7.55 to −4.12)	<0.001
12 months	≤10% decline	124	52.47	54.19	53.14	−1.05 (−2.58 to +0.47)	0.175	+0.67 (−1.10 to +2.44)	0.457
12 months	>10% decline	71	55.21	43.52	48.22	+4.70 (+2.95 to +6.45)	<0.001	−6.99 (−9.20 to −4.78)	<0.001

Values are mean eGFR or mean within-patient changes (95% CI), expressed in mL/min/1.73 m^2^. Changes were calculated as follow-up minus early or follow-up minus baseline eGFR. *p*-values were obtained using two-sided paired *t* tests, with 95% CIs based on the paired differences. Analyses included patients with available measurements at the corresponding follow-up timepoint (259 at 6 months and 195 at 12 months).

## Data Availability

The data underlying this article are not publicly available because they contain information that could compromise participant privacy. De-identified data supporting the findings of this study may be made available from the corresponding author upon reasonable request, subject to institutional approval and applicable data protection regulations.

## Supplementary Materials

The following supporting information can be downloaded at: https://www.mdpi.com/article/10.3390/jcm15208158/s1, Table S1, Characteristics by 12-month eGFR availability; Table S2, Sensitivity analyses of early eGFR decline, Panels A–C. Continuous absolute change, Alternative relative decline thresholds, and baseline eGFR strata; Table S3, Sensitivity analysis excluding extreme early changes, Panels A–B. Multivariable logistic regression, Longitudinal mixed-effects analysis; Table S4A, GEE sensitivity, Panels A–B. Adjusted marginal mean eGFR at observed timepoints, Early assessment–referenced within- and between-group contrasts; Table S4B, Mixed-effects sensitivity analysis restricted to patients with 12-month data, Panels A–B. Adjusted marginal mean eGFR at observed timepoints, Early assessment–referenced within- and between-group contrasts; Table S5, Expanded logistic regression model; Table S6, Timing of early creatinine assessment, Panels A–B. Distribution of sampling intervals, Adjustment for sampling interval; Table S7, ARNI initiation timing and clinical context; Table S8, Model specification sensitivity, Panels A–B. Age modeled continuously and Nonlinearity of baseline eGFR.
