# Supplementary material for: Early Relative eGFR Decline After SGLT2 Inhibitor Initiation in Heart Failure: Clinical Correlates and Pretreatment-Anchored 12-Month Trajectories
Source: J Clin Med. 2026 Oct 20;15(20):8158. doi: 10.3390/jcm15208158 (PMC13706587; doi:10.3390/jcm15208158)
Supplement: Supplementary file 1 [file jcm-15-08158-s001.zip › jcm-4606363-supplementary.pdf]

Table S1. Characteristics by 12-month eGFR availability

Variable	Available (n=195)	Unavailable (n=100)	P value
Age, years	74.6 ± 11.8	74.5 ± 10.4	0.977
Age ≥80 years	77 (39.5%)	31 (31.0%)	0.162
Female sex	58 (29.7%)	31 (31.0%)	0.894
BMI, kg/m²	23.9 ± 4.4	24.1 ± 4.0	0.641
Baseline eGFR	53.5 ± 16.9	57.3 ± 18.1	0.083
LVEF, %	48.0 ± 16.3	48.7 ± 16.2	0.743
HFrEF	60 (30.8%)	31 (31.0%)	0.549
HFmrEF	39 (20.0%)	15 (15.0%)	
HFpEF	96 (49.2%)	54 (54.0%)	
NYHA class III/IV	100 (51.3%)	42 (42.0%)	0.141
NT-proBNP, pg/mL	1441.0 [900.0–2919.5]	1236.5 [681.8–2268.0]	0.143
Hypertension	98 (50.3%)	59 (59.0%)	0.176
Diabetes mellitus	58 (29.7%)	25 (25.0%)	0.415
RAS inhibitor use (excluding ARNI)	49 (25.1%)	28 (28.0%)	0.675
ARNI at SGLT2i initiation	32 (16.4%)	18 (18.0%)	0.745
Beta-blocker use	164 (84.1%)	85 (85.0%)	1
MRA use	116 (59.5%)	55 (55.0%)	0.533
Loop diuretic use	123 (63.1%)	44 (44.0%)	0.002
Tolvaptan use	41 (21.0%)	13 (13.0%)	0.112
Dapagliflozin	115 (59.0%)	56 (56.0%)	0.709
Early decline >10%	71 (36.4%)	39 (39.0%)	0.703
Inpatient SGLT2i initiation	106 (54.4%)	44 (44.0%)	0.11

Data are presented as mean ± SD, median [IQR], or n (%). Patients were classified according to the availability of 12-month eGFR measurements (available, n=195; unavailable, n=100). P values were calculated using Welch's t test for continuous variables, the Mann–Whitney U test for NT-proBNP, and Fisher's exact test for binary variables. The P value for HF phenotype represents an overall comparison of the three HF categories using Pearson's chi-square test.

Table S2. Sensitivity analyses of early eGFR decline

Panel A

Factor	Adjusted β (95% CI), mL/min/1.73 m²	P value
Age ≥80 years	+3.65 (+1.46 to +5.84)	0.001
Female sex	+4.94 (+2.25 to +7.63)	<0.001
Baseline eGFR (per 10 mL/min/1.73 m²)	+2.61 (+1.92 to +3.31)	<0.001
ARNI at SGLT2i initiation	+1.35 (-1.46 to +4.16)	0.346
Loop diuretic use	+0.55 (-1.75 to +2.84)	0.64
Diabetes mellitus	-0.30 (-2.64 to +2.04)	0.803
HFpEF	-2.06 (-4.26 to +0.13)	0.066
Tolvaptan use	+2.56 (-0.76 to +5.87)	0.131
BMI (per 5 kg/m²)	+0.41 (-1.08 to +1.90)	0.591

Panel B

Factor	>10%: OR (95% CI)	P value	>15%: OR (95% CI)	P value	>20%: OR (95% CI)	P value
Age ≥80 years	2.14 (1.21–3.81)	0.009	1.84 (0.99–3.41)	0.054	1.77 (0.85–3.66)	0.125
Female sex	2.51 (1.42–4.44)	0.002	3.03 (1.65–5.57)	<0.001	4.35 (2.13–8.89)	<0.001
Baseline eGFR (per 10 mL/min/1.73 m²)	1.43 (1.21–1.69)	<0.001	1.44 (1.20–1.73)	<0.001	1.58 (1.28–1.96)	<0.001
ARNI at SGLT2i initiation	1.41 (0.71–2.79)	0.32	1.55 (0.74–3.24)	0.241	1.19 (0.48–2.94)	0.713
Loop diuretic use	1.82 (1.03–3.23)	0.039	1.74 (0.93–3.26)	0.082	1.57 (0.74–3.32)	0.238
Diabetes mellitus	0.57 (0.32–1.03)	0.065	0.58 (0.30–1.12)	0.102	0.73 (0.34–1.57)	0.426
HFpEF	0.61 (0.35–1.05)	0.076	0.46 (0.25–0.84)	0.012	0.31 (0.15–0.65)	0.002
Tolvaptan use	1.50 (0.73–3.07)	0.267	1.66 (0.77–3.57)	0.196	2.65 (1.12–6.31)	0.027
BMI (per 5 kg/m²)	1.01 (0.75–1.36)	0.924	0.86 (0.62–1.19)	0.368	0.82 (0.56–1.21)	0.318

Panel A. Absolute eGFR decline
Absolute eGFR decline was defined as baseline minus early eGFR (mL/min/1.73 m²); positive regression coefficients indicate greater eGFR decline. Estimates were derived from multivariable linear regression including the nine covariates used in the primary analysis. Heteroskedasticity-robust standard errors (HC3) were used to calculate 95% CIs and two-sided P values (n=295).
Panel B. Alternative relative decline thresholds
Multivariable logistic regression models were fitted for early relative eGFR decline >10% (110 events), >15% (80 events), and >20% (53 events). Each model included the same nine covariates as the primary analysis. Adjusted odds ratios (ORs), Wald 95% CIs, and two-sided P values are presented. These threshold analyses were exploratory, without adjustment for multiple comparisons. A multivariable model for >30% decline was not fitted because only 19 patients met this threshold.

Table S2. Sensitivity analyses of early eGFR decline

Panel C

Baseline eGFR stratum	n	>10% decline, n (%)	Early change, median [IQR], %	Baseline eGFR, mean ± SD	Early eGFR, mean ± SD	6-month eGFR, mean ± SD (n)	12-month eGFR, mean ± SD (n)
<45	86	24 (27.9%)	-3.0 [-11.3 to 5.3]	33.8 ± 7.3	33.7 ± 9.0	35.6 ± 9.5 (n=79)	35.3 ± 9.9 (n=61)
45–59	92	39 (42.4%)	-4.3 [-17.9 to 3.1]	52.5 ± 4.3	48.9 ± 10.6	49.8 ± 10.3 (n=81)	50.8 ± 11.2 (n=61)
≥60	117	47 (40.2%)	-6.6 [-18.1 to -0.3]	71.9 ± 9.2	65.0 ± 11.8	66.8 ± 11.5 (n=99)	65.2 ± 12.9 (n=73)

Panel C. Baseline eGFR strata

Patients were stratified by baseline eGFR (<45, 45–<60, and ≥60 mL/min/1.73 m²). Early relative eGFR change is presented as median [IQR] (%); eGFR measurements at baseline, early assessment, 6 months, and 12 months are presented as mean ± SD (mL/min/1.73 m²). Follow-up sample sizes vary according to measurement availability.

Table S3. Sensitivity analysis excluding extreme early changes

Panel A

Factor	Primary OR (95% CI)	P value	Excluding 6 extremes: OR (95% CI)	P value
Age ≥80 years	2.14 (1.21–3.81)	0.009	2.07 (1.16–3.70)	0.013
Female sex	2.51 (1.42–4.44)	0.002	2.47 (1.38–4.40)	0.002
Baseline eGFR (per 10 mL/min/1.73 m²)	1.43 (1.21–1.69)	<0.001	1.40 (1.18–1.66)	<0.001
ARNI at SGLT2i initiation	1.41 (0.71–2.79)	0.32	1.36 (0.68–2.72)	0.381
Loop diuretic use	1.82 (1.03–3.23)	0.039	1.85 (1.04–3.29)	0.036
Diabetes mellitus	0.57 (0.32–1.03)	0.065	0.55 (0.30–1.00)	0.05
HFpEF	0.61 (0.35–1.05)	0.076	0.61 (0.35–1.07)	0.084
Tolvaptan use	1.50 (0.73–3.07)	0.267	1.51 (0.73–3.12)	0.262
BMI (per 5 kg/m²)	1.01 (0.75–1.36)	0.924	0.98 (0.73–1.32)	0.892

Panel B

Adjusted eGFR contrast	Δ (95% CI), mL/min/1.73 m²	P value
≤10%: 6 months − early	-1.31 (-2.48 to -0.14)	0.028
≤10%: 12 months − early	-0.73 (-2.02 to +0.55)	0.264
>10%: 6 months − early	+5.98 (+4.47 to +7.48)	<0.001
>10%: 12 months − early	+4.76 (+3.08 to +6.43)	<0.001
Between-group Δ: 6 months	+7.29 (+5.38 to +9.19)	<0.001
Between-group Δ: 12 months	+5.49 (+3.38 to +7.60)	<0.001

Panel A. Multivariable logistic regression
Six patients with an absolute early relative eGFR change ≥50% were excluded in this sensitivity analysis (primary cohort, n=295; restricted cohort, n=289). Adjusted ORs, Wald 95% CIs, and two-sided P values were obtained from logistic regression models including the same nine covariates as the primary analysis.
Panel B. Longitudinal mixed-effects analysis
Adjusted contrasts were estimated using a linear mixed-effects model restricted to the 289 patients remaining after exclusion of extreme changes. The model included early decline group, assessment time, their interaction, and the covariates used in the primary longitudinal analysis. Estimates represent changes from early assessment within each group or between-group differences in these changes, expressed in mL/min/1.73 m².
Six within- and between-group contrasts are presented with 95% CIs and two-sided P values without multiplicity adjustment.

Table S4A. GEE sensitivity analysis

Panel A. Adjusted marginal mean eGFR at observed timepoints

Early decline group	Assessment	Adjusted eGFR	Lower 95% CI	Upper 95% CI	SE
≤10% decline	Early (within 45 days)	55.58	54.69	56.47	0.46
>10% decline	Early (within 45 days)	42.93	41.67	44.18	0.64
≤10% decline	6 months	54.11	52.80	55.42	0.67
>10% decline	6 months	49.20	47.66	50.73	0.78
≤10% decline	12 months	54.81	53.27	56.35	0.79
>10% decline	12 months	47.97	46.14	49.79	0.93

Panel B. Early assessment–referenced within- and between-group contrasts

Contrast	Estimate Δ	Lower 95% CI	Upper 95% CI	P value	SE
≤10%: 6 months − early	-1.47	-2.71	-0.23	0.021	0.63
≤10%: 12 months − early	-0.77	-2.21	+0.67	0.292	0.73
>10%: 6 months − early	+6.27	+4.69	+7.85	<0.001	0.80
>10%: 12 months − early	+5.04	+3.31	+6.77	<0.001	0.88
Between-group Δ: 6 months	+7.74	+5.73	+9.74	<0.001	1.02
Between-group Δ: 12 months	+5.81	+3.57	+8.06	<0.001	1.14
Global group × time interaction					
Wald χ^2	58.14 (df=2)		P <0.001		

Generalized estimating equations (GEE) with an exchangeable working correlation structure and sandwich-robust standard errors were applied to 749 observed eGFR measurements from 295 patients. Models adjusted for baseline eGFR, age ≥80 years, female sex, ARNI use at SGLT2 inhibitor initiation, and loop diuretic use. Adjusted marginal means were standardized to the covariate means of the 295 patients. The global group-by-time interaction was evaluated using a Wald test. Between-group contrasts represent differences in changes from early assessment (>10% decline group minus ≤10% decline group). Six within- and between-group contrasts are presented with 95% confidence intervals and two-sided P values without multiplicity adjustment.

Table S4B. Mixed-effects sensitivity analysis restricted to patients with 12-month data

Panel A. Adjusted marginal mean eGFR at observed timepoints

Early decline group	Assessment	Adjusted eGFR	Lower 95% CI	Upper 95% CI	SE
≤10% decline	Early (within 45 days)	54.76	53.37	56.16	0.71
>10% decline	Early (within 45 days)	42.52	40.66	44.37	0.95
≤10% decline	6 months	52.67	51.24	54.09	0.73
>10% decline	6 months	48.54	46.64	50.44	0.97
≤10% decline	12 months	53.71	52.32	55.10	0.71
>10% decline	12 months	47.22	45.36	49.07	0.95

Panel B. Early assessment–referenced within- and between-group contrasts

Contrast	Estimate Δ	Lower 95% CI	Upper 95% CI	P value	SE
≤10%: 6 months − early	-2.10	-3.47	-0.72	0.003	0.70
≤10%: 12 months − early	-1.05	-2.39	+0.29	0.123	0.68
>10%: 6 months − early	+6.03	+4.20	+7.85	<0.001	0.93
>10%: 12 months − early	+4.70	+2.93	+6.47	<0.001	0.90
Between-group Δ: 6 months	+8.12	+5.84	+10.41	<0.001	1.17
Between-group Δ: 12 months	+5.76	+3.53	+7.98	<0.001	1.13

Global group × time interaction		
ML likelihood ratio χ^2	49.56 (df=2)	P <0.001

The analysis included 195 patients with available baseline, early, and 12-month eGFR measurements (570 observations); 180 patients also had 6-month measurements. A linear mixed-effects model with a patient-specific random intercept was fitted using restricted maximum likelihood (REML), adjusting for the same covariates as the primary longitudinal model. Adjusted marginal means were standardized to the covariate means of the 195-patient subgroup. The global group-by-time interaction was assessed by a likelihood-ratio test comparing models fitted using maximum likelihood. Between-group contrasts represent differences in changes from early assessment (>10% decline group minus ≤10% decline group). Six within- and between-group contrasts are presented with 95% confidence intervals and two-sided P values without multiplicity adjustment.

Table S5. Expanded logistic regression model

Factor	Primary adjusted OR (95% CI)	P value	Expanded adjusted OR (95% CI)	P value
Age ≥80 years	2.14 (1.21–3.81)	0.009	2.15 (1.19–3.88)	0.011
Female sex	2.51 (1.42–4.44)	0.002	2.79 (1.55–5.04)	<0.001
Baseline eGFR (per 10 mL/min/1.73 m²)	1.43 (1.21–1.69)	<0.001	1.38 (1.16–1.65)	<0.001
ARNI at SGLT2i initiation	1.41 (0.71–2.79)	0.32	1.79 (0.81–3.95)	0.147
Loop diuretic use	1.82 (1.03–3.23)	0.039	1.18 (0.62–2.24)	0.621
Diabetes mellitus	0.57 (0.32–1.03)	0.065	0.58 (0.31–1.08)	0.087
HFpEF	0.61 (0.35–1.05)	0.076	0.72 (0.40–1.27)	0.253
Tolvaptan use	1.50 (0.73–3.07)	0.267	1.24 (0.59–2.61)	0.571
BMI (per 5 kg/m²)	1.01 (0.75–1.36)	0.924	1.09 (0.80–1.48)	0.588
NYHA class III/IV	—	—	1.93 (0.97–3.83)	0.062
MRA use	—	—	1.87 (1.02–3.43)	0.044
Dapagliflozin vs empagliflozin	—	—	1.70 (0.96–3.02)	0.071

The expanded multivariable logistic regression model included the nine covariates from the primary model, with additional adjustment for NYHA class III/IV, MRA use, and SGLT2 inhibitor type (dapagliflozin versus empagliflozin). Adjusted ORs, Wald 95% CIs, and two-sided P values are presented. ARNI exposure was defined as use at SGLT2 inhibitor initiation (n=50). Results from the expanded model are exploratory and should not be interpreted as causal treatment effects.

Table S6. Timing of early creatinine assessment

Panel A

Sampling interval	n	Early decline >10%, n	Early decline >10%, %	≤10% decline, n	Days, median [IQR]
10–13 days	5	2	40.0	3	12 [12–13]
14–20 days	61	18	29.5	43	17 [15–18]
21–29 days	134	48	35.8	86	25 [22–27.8]
30–45 days	95	42	44.2	53	33 [30–36]
Total	295	110	37.3	185	25 [21–30]

Panel B

Factor	All 295: OR (95% CI)	P value	Days 14–45: OR (95% CI)	P value
Age ≥80 years	2.42 (1.34–4.36)	0.003	2.51 (1.38–4.57)	0.003
Female sex	2.83 (1.57–5.08)	<0.001	2.85 (1.58–5.16)	<0.001
Baseline eGFR (per 10 mL/min/1.73 m²)	1.51 (1.26–1.80)	<0.001	1.49 (1.25–1.79)	<0.001
ARNI at SGLT2i initiation	1.00 (0.48–2.07)	0.991	0.96 (0.46–1.99)	0.909
Loop diuretic use	1.79 (1.01–3.17)	0.048	1.73 (0.97–3.10)	0.063
Diabetes mellitus	0.50 (0.27–0.93)	0.028	0.48 (0.26–0.89)	0.02
HFpEF	0.63 (0.36–1.10)	0.104	0.63 (0.36–1.11)	0.11
Tolvaptan use	1.50 (0.73–3.11)	0.272	1.41 (0.67–2.94)	0.363
BMI (per 5 kg/m²)	1.04 (0.77–1.41)	0.785	1.07 (0.79–1.45)	0.646
Timing to early sample (per 7 days)	1.48 (1.10–1.99)	0.009	1.55 (1.14–2.11)	0.005

Panel A. Distribution of sampling intervals
The early assessment was defined as the first eligible serum creatinine measurement on days 10–45 after SGLT2 inhibitor initiation; measurements obtained on days 1–9 were not eligible. Sampling occurred 10–42 days after initiation (median 25 days [IQR 21–30]). Data are presented as n (%), unless otherwise indicated. Early relative eGFR decline >10% was assessed at the corresponding first follow-up measurement.
Panel B. Adjustment for sampling interval
Multivariable logistic regression included the nine covariates from the primary model and the time from SGLT2 inhibitor initiation to early assessment, modeled continuously per 7 days. Analyses were performed in the full cohort (n=295) and after excluding five patients assessed within 10–13 days (restricted cohort, n=290). Adjusted ORs, Wald 95% CIs, and two-sided P values are presented. Associations with sampling interval may reflect clinical scheduling and patient characteristics and should not be interpreted causally.

Table S7. ARNI initiation timing and clinical context

ARNI exposure category	n	Early decline >10%, n	Early decline >10%, %	Inpatient SGLT2i initiation, n	Inpatient, %	ARNI start minus SGLT2i start, median [IQR], days
No ARNI recorded	169	53	31.4	82	48.5	—
ARNI already at SGLT2i start	50	20	40.0	1	2.0	−24 [−28.5 to −21]
ARNI initiated after SGLT2i	76	37	48.7	67	88.2	4 [3–5]
Total	295	110	37.3	150	50.8	—

ARNI exposure was classified according to its timing relative to SGLT2 inhibitor initiation: no ARNI recorded (n=169), ARNI use already established at initiation (n=50), or ARNI initiated subsequently (n=76). Timing was calculated as ARNI initiation date minus SGLT2 inhibitor initiation date; negative values indicate earlier ARNI initiation. Only ARNI use already established at SGLT2 inhibitor initiation was considered baseline ARNI exposure in the primary regression models. Data are presented as n (%), or median [IQR] where indicated.

Table S8. Model specification sensitivity

Panel A

Factor	Adjusted OR (95% CI)	P value
Age (per 10 years)	1.45 (1.11–1.90)	0.007
Female sex	2.35 (1.32–4.18)	0.004
Baseline eGFR (per 10 mL/min/1.73 m²)	1.47 (1.23–1.75)	<0.001
ARNI at SGLT2i initiation	1.39 (0.71–2.75)	0.338
Loop diuretic use	1.81 (1.02–3.21)	0.042
Diabetes mellitus	0.59 (0.33–1.08)	0.086
HFpEF	0.60 (0.35–1.04)	0.069
Tolvaptan use	1.49 (0.73–3.05)	0.275
BMI (per 5 kg/m²)	1.11 (0.82–1.52)	0.499

Panel B

Model / comparison	n	AIC	LR χ^2 (df)	P value
Linear eGFR	295	369.92	—	—
4-knot RCS eGFR	295	372.53	—	—
Nonlinearity LR test	295		1.39 (2)	0.499

Restricted cubic spline knots		
Percentile	eGFR knot	mL/min/1.73 m²
5	26.21	
35	47.69	
65	61.92	
95	81.06	

Panel A. Age modeled continuously
The primary multivariable logistic regression model was refitted with age entered as a continuous variable per 10-year increment instead of the dichotomous age ≥80 years variable. The remaining eight covariates were unchanged. Adjusted ORs, Wald 95% CIs, and two-sided P values are presented (n=295).
Panel B. Nonlinearity of baseline eGFR
The linear association between baseline eGFR and early relative decline >10% was compared with a restricted cubic spline (RCS) model using four knots at the 5th, 35th, 65th, and 95th percentiles of baseline eGFR. Both models included the same remaining covariates. Nonlinearity was evaluated using a likelihood-ratio test comparing the spline and linear models (2 degrees of freedom). Model fit was summarized using the Akaike information criterion (AIC). A nonsignificant test does not establish linearity.
